# Diagnostic performance of the metagenomic next-generation sequencing in lung biopsy tissues in patients suspected of having a local pulmonary infection

**DOI:** 10.1186/s12890-022-01912-4

**Published:** 2022-03-29

**Authors:** Shan-shan Su, Xue-bing Chen, Ling-ping Zhou, Peng-cheng Lin, Jun-jie Chen, Cheng-shui Chen, Qing Wu, Jun-ru Ye, Yu-ping Li

**Affiliations:** 1 The Key Laboratory of Interventional Pulmonology of Zhejiang Province, Department of Pulmonary and Critical Care Medicine, The First Affiliated Hospital of Wenzhou Medical University, South Baixiang, Ouhai District, Wenzhou, 325015 Zhejiang People’s Republic of China; 2 Medical Record Statistics Room, Medical Quality Control Department, The First Affiliated Hospital of Wenzhou Medical University, Wenzhou, 325015 Zhejiang People’s Republic of China; 3 The Center of Laboratory and Diagnosis, The First Affiliated Hospital of Wenzhou Medical University, Wenzhou, 325015 Zhejiang People’s Republic of China

**Keywords:** Local pulmonary infections, CT-guided lung biopsy, Metagenomic next-generation sequencing, Conventional examinations, Lung abscess

## Abstract

**Purpose:**

This study aims to evaluate the diagnostic application and performance of the metagenomic next-generation sequencing (mNGS) in patients suspected of local pulmonary infection by comparing it to the traditional pathogen detection methods in lung tissue specimens obtained by a computerized tomography-guided biopsy (CT-guided biopsy).

**Methods:**

We retrospectively reviewed patients, admitted to the First Affiliated Hospital of Wenzhou Medical University, China from May 2018 to December 2020, who were suspected of local pulmonary infection. All cases received a CT-guided lung biopsy, tissue samples were sent both for conventional examinations (CE) and mNGS tests. The sensitivity and specificity of the two diagnostic approaches were compared.

**Results:**

106 patients enrolled, 76 patients were diagnosed with a pulmonary infection. Among 49 patients with identified pathogens, CE confirmed pathogenic infections in 32 cases. *Mycobacterium* spp. and fungi accounted for 37.5% (12/32) and 28.1% (9/32), respectively, with bacteria 34.4% (11/32). The mNGS examination detected extra pathogenic microorganisms in 22 patients that were consistent with the patients' clinical and radiographic pictures. The sensitivity of mNGS was 53.9% vs. 42.1% for the CE, while the specificity was 56.7% versus 96.7%. For detection rate, mNGS was significantly superior to CE in bacterial (96.3% vs. 40.7%, *p *< 0.05), and mixed infections (100% vs. 50%, *p* < 0.05), but inferior to CE in fungal (60% vs. 90%, *p* > 0.05) and *Mycobacterium* spp. infections (66.7% vs. 100%, *p* > 0.05) with no significant difference. Among 31 cases diagnosed with lung abscess, the diagnostic performance of the detection rate was 67.7% (21/31) in favour of mNGS compared to 29.0% (9/31) for CE (*p* < 0.05). Most polymicrobial infections were induced by anaerobic species that coexisted with *Streptococcus constellatus*. And *Klebsiella pneumoniae* was the most common isolated monomicrobial infection.

**Conclusions:**

The most commonly detected causative pathogens for local pulmonary infections were bacteria, *Mycobacterium* spp. and fungi. Compared with the CE, the advantages of mNGS in the pathogens detection lie in the discovery of bacterial and mixed infections, as well as in the detection of lung abscess. Conversely, mNGS is not good enough to be recommendable for the detection of *Mycobacterium* spp. and fungi.

**Supplementary Information:**

The online version contains supplementary material available at 10.1186/s12890-022-01912-4.

## Introduction

Pulmonary local infections often manifest as a consolidation or mass in the lung, sometimes with a cavity in computerized tomography (CT) scans. These pathological conditions can be due to inadequately treated or atypical infections, such as tuberculosis, fungal infections, chronic or a subacute lung abscess [1], etc. Bronchoscopy, CT or ultrasound-guided percutaneous lung biopsy are the most common techniques used to obtain a lung biopsy to identify the accurate aetiology of the pulmonary diseases [2, 3]. Histopathological diagnosis and microbiological culture of lung tissues are the gold standards for identifying the causative pathogens and to confirm pulmonary infectious diseases. However, these conventional methods are relatively insensitive and time-consuming. Data show that the conventional methods of CT-guided percutaneous lung biopsies, revealed the causative organism in 32.5% of biopsies (114/351) [4]. For lung abscess, the sensitivity of conventional culture examination is limited to the identification of anaerobes for suggesting empirical antibiotic therapy [4]. And studies about percutaneous transthoracic needle biopsy for infectious diseases to identify pathogen are mostly focused on immunocompromised patients, data on those immunocompetent patients are limited. In recent years, the metagenomic next-generation sequencing (mNGS) has been used in clinical practice for pathogen detection in a variety of clinical specimens, such as cerebrospinal fluid, blood and bronchoalveolar lavage fluid (BALF) [5–7]. mNGS has shown its advantages for the diagnosis of mixed infections and infections due to difficult-to-culture bacteria. However, reports on the use of mNGS in local pulmonary infections applied to lung biopsy tissues yet remain scarce and need further studies.

Here, we aim to evaluate the diagnostic performance of mNGS in patients suspected of having a local pulmonary infection by comparing the traditional pathogen detection methods in specimens of lung tissue obtained by a CT-guided lung biopsy. In addition, we also evaluate the diagnostic performance of mNGS in pathogens detection in lung abscess and compare it to the conventional examinations (CE). This study received approval from the Ethics Committee of the First Affiliated Hospital of Wenzhou Medical University (No. 2020-111) and was conducted following the Declaration of Helsinki (as revised in 2013).

## Material and methods

### Study population and procedures

We retrospectively reviewed cases who were suspected of having local pulmonary infections, admitted to the Department of Pulmonary and Critical Care Medicine of the First Affiliated Hospital of Wenzhou Medical University, China from May 2018 to December 2020. All cases received a CT-guided lung biopsy and lung tissues were sent both for CE and mNGS tests. Baseline data were collected from the electronic medical records of the patients, including demographic characteristics, comorbidities and results of CE and mNGS tests. CE included bacterial, fungal and acid-fast bacilli smear of sputum, BALF (when present) or lung biopsy specimen; culture of blood, sputum, BALF or lung biopsy specimen, polymerase chain reactions (PCRs) for detection of influenza A/B virus*,* Cytomegalovirus (CMV), *Mycoplasma *spp., and *Chlamydia *spp*.,* and GeneXpert TB PCR for *Mycobacterium tuberculosis*; pathological testing of lung biopsy specimen, such as haematoxylin and eosin staining, Ziehl–Neelsen, Grocott-Gomori's (or Gömöri) methenamine silver staining (GMS), and periodic acid-Schiff (PAS) staining; Serological tests included: *Cryptococcus* capsular polysaccharide antigen (CrAg) detection, (1,3)-β-d-glucan test (G test), galactomannan test (GM test) and *Chlamydia pneumoniae* and *Mycoplasma pneumoniae* detection by serological antibody detection. Results of histopathology and radiological features were collected at the same time. The follow-up information of patients was obtained on regular clinic visits.

### CT—guided lung biopsy

Under the guidance of multi-detector row computed tomography (CT), biopsies were performed using a coaxial technique with an 18-gauge thin-wall coaxial introducer needle. Core biopsies were performed using a 19-gauge automated cutting needle biopsy gun (Fine Core Biopsy Needle; Nagano, Gyoda-City, Saitama, Japan). After core biopsies, needle aspiration was performed by connecting a 10 mL syringe to a thin-wall outer needle, moving up and down slightly and drawing 2–5 mL (according to the size of the abnormal lesion) of secretion from the abnormal lesion. The specimens were sent to a microbiological laboratory for bacterial and fungal smear and culture tests, acid-fast stain and GeneXpert TB PCR specific for *M. tuberculosis*. Lung tissue samples were also sent to the Pathology department for a histopathology examination. At the end of the procedure, a CT scan was performed for a repeated examination, then patients were sent back to the ward.

### Metagenomic next-generation sequencing—mNGS

The lung tissues were ground to obtain tissue homogenates. 1.5 mL microcentrifuge tube with 0.7 mL lysis buffer and pieces of tissue sample and 1 g 0.5 mm glass bead (Sigma-Aldrich, USA) were attached to a horizontal platform on a vortex mixer (VORTEX-GENIE 2 VORTEX MIXER 12, Scientific Industries, USA) and agitated vigorously at 2800-3200RPM for 30 min at room temperature. 0.3 mL sample was separated into a new 1.5 mL microcentrifuge tube, DNA was extracted according to the steps of TIANampDNA extraction kit (DP316, Tiangen biochemical Technology Co., China).

The DNA library was constructed by DNA fragmentation, DNA end-repair, splice connection and PCR amplification. Agilent 2100 (Agilent, USA) was used to control the quality of the DNA library. BGISEQ-50 sequencing platform (BGI, China) was used for sequencing. To maintain the high quality of the sequencing data, the low-quality readings with a length less than 35 bp were removed. Through the application of BWA alignment [8], the data of human reference genome sequences in high-quality data were removed. The rest of the data were classified and sorted by removing low-complexity sequences and comparing them with four microbial genome databases, including bacteria, fungi, viruses and parasites.

### Diagnostic criteria

Conventional examination pathogenic diagnosis was made following one of the criteria like a) positive culture result of lung tissue or needle aspiration; b) positive GeneXpert TB PCR result of tissue sample DNA or sputum, or BALF; c) positive pathogen finding or the presence of granulomas related to infections, detected with haematoxylin and eosin, Ziehl–Neelsen, GMS, and PAS staining for examination of tissue pathology. The potential pathogen was considered detected by mNGS if it met one of the following criteria: (a) the same pathogen as in the conventional examination has been identified; (b) more than 30% of relative abundance at the bacterial genus level; (c) at least one unique read for *M. tuberculosis complex* (MTBC); (d) when only mNGS identified the pathogen, especially when the CE was strictly negative, we considered it as a potential pathogen when mNGS results were in accordance with the patient’s clinical features, laboratory abnormal results, and moreover, when patient’s condition improved after empirical antibiotic treatment, as introduced in the previous studies [5, 9, 10].

Lung abscess was defined as a circumscribed area of pus or necrosis in the lung parenchyma, which led to a cavity, an air-fluid level or low density inside the lesion [11].

Criteria of immunocompromised status were defined as any of the followings: (1) a long-term therapy with steroids (> 0.3 mg/kg/days of prednisone, equivalent for ≥ 3 weeks) or other immunosuppressant drugs; (2) haematological malignancy; (3) solid-organ transplant receipt during the last 6 months; (4) recent chemotherapy during the last month; (5) inherited or acquired severe immunodeficiency or human immunodeficiency virus (HIV) infection [12].

The final clinical diagnosis was made by two independent clinicians with pooled analysis of the clinical, radiographic, laboratory and conventional microbiological examinations, mNGS results and histopathologic examination results. Clinical diagnoses were finally classified into 3 groups: (1) a pulmonary infectious disease with a definite pathogen, (2) a pulmonary infectious disease with suspicion of infection with no identified pathogen, and (3) a non-infectious pulmonary disease.

### Data analysis

Statistical analyses were conducted using the SPSS statistical package 12.0 software. Quantitative data were expressed as mean ± standard deviation or medians (25th, 75th percentiles). The count data were expressed as a percentage of the number of cases. Comparisons were performed using the Pearson’s Chi-square test, Fishers exact Chi-square test and the McNemar test. All *p* values were considered significant at *p* < 0.05. The concordance analysis was estimated using the Kappa test (kappa ≤ 0.40, poor agreement; kappa ≤ 0.60, moderate agreement; kappa ≤ 0.8, good agreement; and kappa > 0.80, excellent agreement).

## Results

### Patients characteristics

**Fig. 1:**
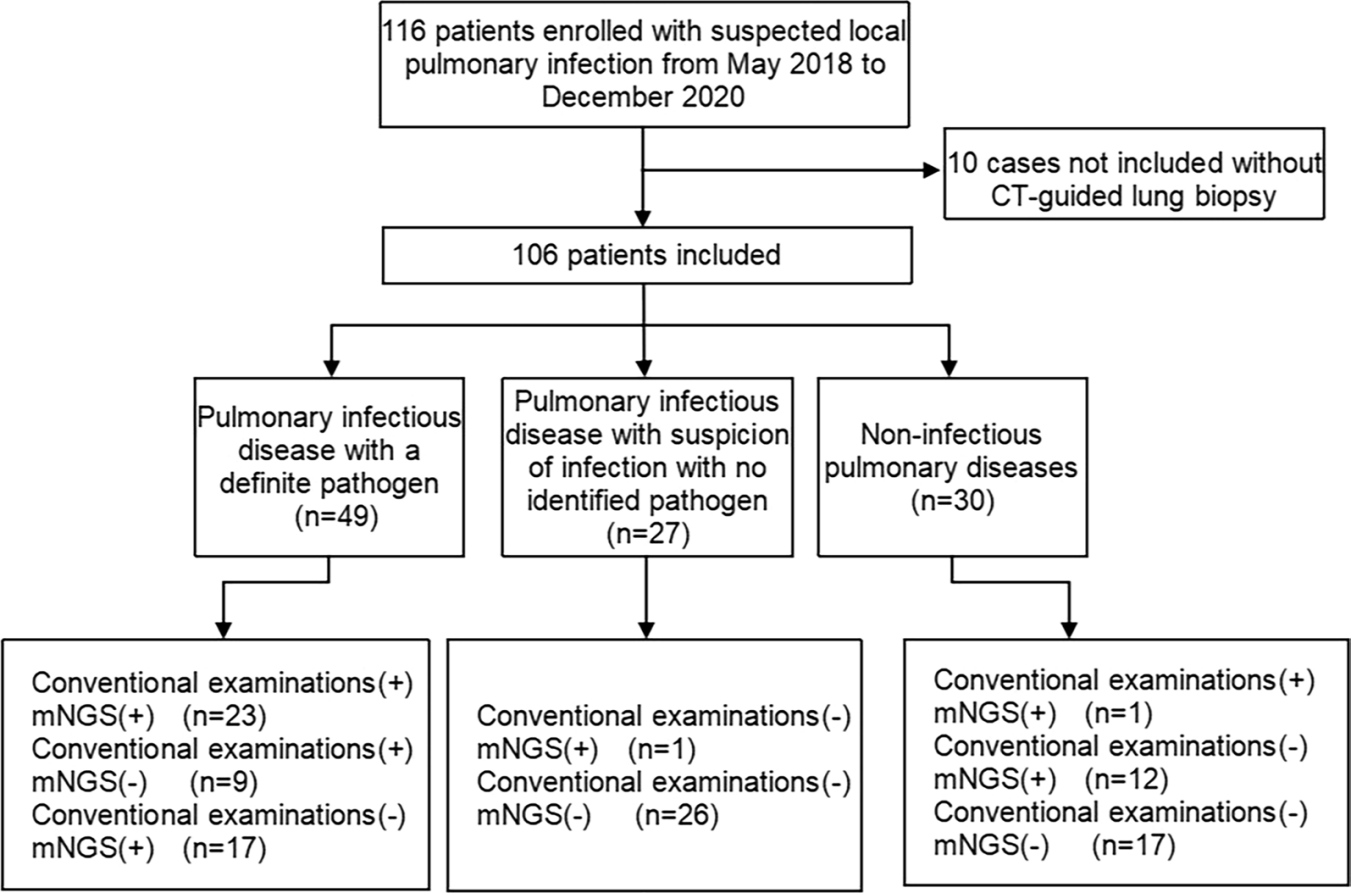
The flow chart for enrollment

In the current study, 106 patients were enrolled. 76 (71.7%) were finally diagnosed with a pulmonary infectious disease (Fig. 1). Of them, 49 (64.5%) patients had a microbiologically confirmed pathogenic infection, 27 patients (35.5%) were diagnosed with presence of infection with no identified pathogen. Among the 76 patients, the main symptoms included cough, expectoration, fever, haemoptysis and chest pain. The symptoms duration before admission was estimated as less than 1 month in 56.6%, 1–3 months in 14.5% and more than 3 months in 28.9% of the patients. The most common underlying disease was hypertension, followed by diabetes mellitus and immunosuppressive status. Radiologic appearance in the CT images of these 76 cases detected either a nodule or mass in the lung (77.6%); a consolidation (32.9%), necrosis (25%), pleural effusion (19.7%), or a mediastinal lymph node enlargement (23.7%). Among the total number of all 106 cases, enrolled in the study, 14 patients had the condition of immunosuppressed status, with 10 cases considered as a pulmonary infectious disease, of which 7 cases had a definitely proven presence of pathogens. Detailed data are listed in Table 1.

**Table 1:** Baseline data of cases suspected of having a local pulmonary infection

Characteristics	Pulmonary infectious disease with a definite pathogen (N = 49)	Pulmonary infectious disease with suspicion of infection with no identified pathogen (N = 27)	Non-infectious pulmonary diseases (N = 30)
Gender, N (%)			
Male	36 (73.5)	20 (74.1)	20 (66.7)
Female	13 (26.5)	7 (25.9)	10 (33.3)
Age (years)	60 (46,65)	61 (50,71)	61 (50,71)
Symptom, N (%)			
Cough	35 (71.4)	20 (74.1)	21 (70.0)
Expectoration	25 (51.0)	17 (63.0)	13 (43.3)
Hemoptysis	12 (24.5)	5 (18.5)	3 (10.0)
Dyspnea	7 (14.3)	3 (11.1)	10 (33.3)
Fever	14 (28.6)	9 (33.3)	6 (20.0)
Chest pain	13 (26.5)	3 (11.1)	7 (23.3)
Other symptoms	5 (10.2)	3 (11.1)	5 (16.7)
Asymptomatic	5 (10.2)	2 (7.4)	3 (10.0)
Symptom duration before admission (days)			
≤ 1 month, N (%)	27 (55.1)	16 (59.3)	17 (56.7)
> 1 month, ≤ 3 months, N (%)	7 (14.3)	4 (14.8)	5 (16.7)
> 3 months, ≤ 1 year, N (%)	10 (20.4)	4 (14.8)	4 (13.3)
> 1 year, N (%)	5 (10.2)	3 (11.1)	4 (13.3)
Underlying disease, N (%)			
Hypertension	15 (30.6)	9 (33.3)	10 (33.3)
Diabetes mellitus	8 (16.3)	7 (25.9)	7 (23.3)
Malignant tumor	1 (2.0)	2 (7.4)	4 (13.3)
Immunosuppressive status	7 (14.3)	3 (11.1)	4 (13.3)
Other diseases	2 (4.1)	2 (7.4)	1 (3.3)
No underlying disease, N (%)	29 (59.2)	15 (55.6)	15 (50.0)
Laboratory examination			
White blood cell count (× 10^9^/L)	7.5 (5.7,12.0)	8.7 (6.1,12.7)	8.7 (6.4,10.6)
Percentage of neutrophils (%)	67.7 (59.2,76.1)	69.8 (64.5,84.2)	68.9 (63.0,78.4)
Percentage of lymphocytes (%)	22.1 (11.6,30.3)	20.3 (9.2,24.3)	16.7 (10.1,25.1)
Lobes of the lungs involved, N (%)			
Single lobe	20 (40.8)	9 (33.3)	5 (16.7)
Multi lobes	29 (59.2)	18 (66.7)	25 (83.3)
Imaging features, N (%)			
Consolidation	16 (32.7)	9 (33.3)	11 (36.7)
Nodule or mass	38 (77.6)	21 (77.8)	25 (83.3)
Cavity	12 (24.5)	5 (18.5)	8 (26.7)
Necrosis	14 (28.6)	5 (18.5)	4 (13.3)
Interstitial lesion	2 (4.1)	0	2 (6.7)
Pleural effusion	11 (22.4)	4 (14.8)	8 (26.7)
Mediastinal lymph node enlargement	14 (28.6)	4 (14.8)	12 (40.0)

### Pathogen distribution

#### CE pathogenic diagnostics

Among the 49 patients with identified pathogens, 32 cases were diagnosed by CE. In these 32 cases, *Mycobacterium *spp*.* accounted for 37.5% (12/32), which consisted of 10 cases with *M. tuberculosis* infection (31.3%, 10/32), and 2 cases with the *nontuberculous* *Mycobacteria* (6.3%, 2/32). Fungal infections were confirmed in 28.1% (9/32) of the patients, with the following detected fungi: *Cryptococcus neoformans* (n = 4), *Aspergillus fumigatus* (n = 3), and *Penicillium marneffei* (n = 2), respectively. 34.4% (11/32) patients were diagnosed with bacterial infections, among which *Pseudomonas aeruginosa* (n = 1), *Streptococcus constellatus* (n = 4), *Actinomycetes* (n = 2), *Nocardia* (n = 1), *Legionella* (n = 1), *Streptococcus pneumoniae* (n = 1) and *Fusobacterium nucleatum* (n = 1).

#### mNGS pathogenic diagnostics

**Fig. 2:**
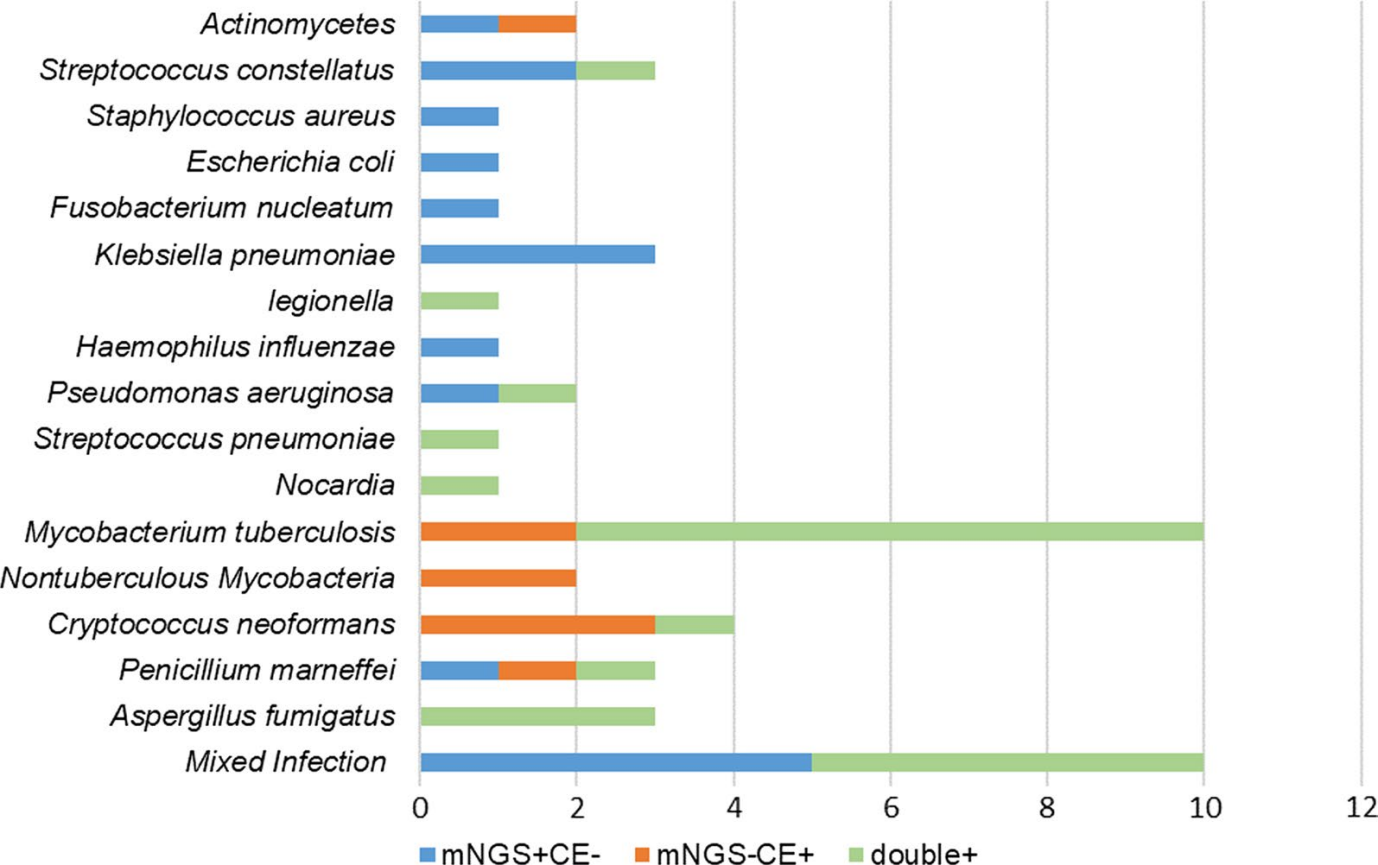
Distribution of pathogens identified in local pulmonary infection with mNGS or conventional examinations. Conventional examination only found single pathogen in 50% (5/10) of polymicrobial infectious cases

By applying the mNGS technique we have detected extra pathogenic microorganisms in 22 patients that were consistent with the patient’s clinical and radiographic pictures (Fig. 2). Among them, polymicrobial infections were found in 10 cases: *Porphyromonas endodontalis*, n = 5, *Treponema lecithinolyticum*, n = 3, *Treponema denticola*, n = 5, *Parvimonas micr*a, n = 4, *Capnocytophaga sputigena*, n = 1, *Actinomycetes*, n = 1, *Streptococcus constellatu*s, n = 4, *Fusobacterium nucleatum*, n = 1. However, CE only detected a single pathogen in half (5/10) of the polymicrobial infectious cases, namely *Streptococcus constellatus*, n = 3, *Actinomycetes,* n = 1, and *Fusobacterium nucleatum*, n = 1. Compared with the conventional diagnostic tests for pathogens, mNGS analysis yielded false-negative results in 9 samples: *M. tuberculosis*, n = 2, *Nontuberculous mycobacteria,* n = 2, *Cryptococcus neoformans*, n = 3, *Penicillium marneffe*i, n = 1 and *Actinomycetes*, n = 1.

An additional file shows more details about these 76 cases (see Additional file 1).

#### Comparison between the detection rates of CE and mNGS techniques

**Fig. 3:**
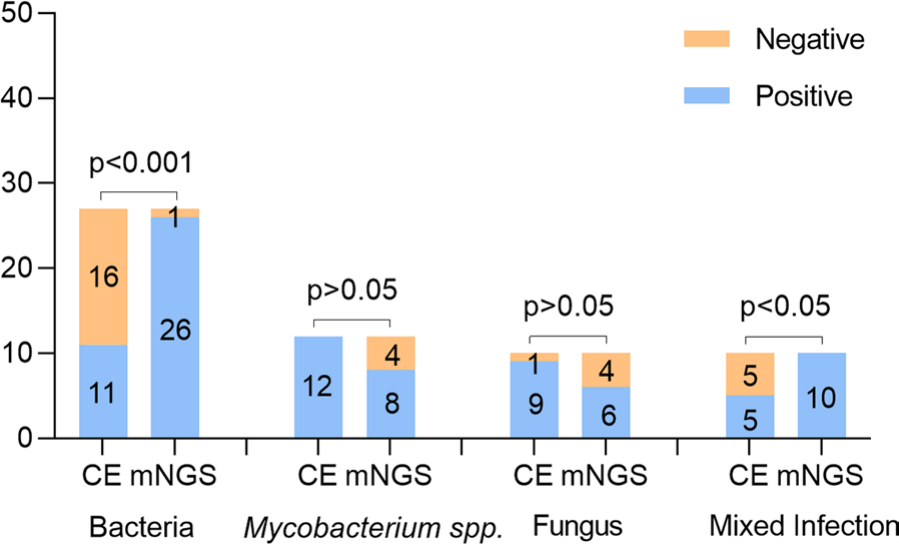
Comparison of CE and mNGS test for the different classes of pathogens. *Notes* The number of positive samples (y-axis) for pairwise mNGS and CE is plotted against the bacteria, *Mycobacterium* spp. fungus and mixed infection groups (x-axis). mNGS: metagenomic next-generation sequencing; CE: conventional examinations

We have further compared the pathogenic detection rate between mNGS and CE and found that the mNGS was significantly superior to the CE for detection rate of bacterial (96.3% vs. 40.7%, *p* < 0.05) and mixed infections (100% vs. 50%, *p* < 0.05), but proved inferior in fungal (60% vs. 90%, *p* > 0.05) and *Mycobacterium *spp*.* infections with no statistically significant difference (66.7% vs. 100%, *p* > 0.05) (see Fig. 3).

#### Pulmonary infectious disease with suspicion of infection with no identified pathogen

In all 27 cases that were finally diagnosed as a pulmonary infectious disease, neither CE nor mNGS identified the presence of any infectious microorganism. The histopathology results and clinical diagnosis of these 27 cases are shown in Table 2.

**Table 2:** Histopathological results of patients with pulmonary infectious disease with suspicion of infection with no identified pathogen

Patient ID	Symptom duration before admission (days)	Age (years)	Histopathology results	Clinical diagnosis
P8	60	50	Inflammatory cells	Lung abscess
P9	90	66	Inflammatory cells with few fibrosis	Lung abscess
P12	10	48	Inflammatory cells with few fibrosis	Pulmonary infection
P13	10	23	Inflammatory lesion	Pulmonary infection
P15	30	71	Organizing pneumonia	Pulmonary infection
P27	700	71	Inflammatory cells with necrosis	Lung abscess
P32	10	67	Inflammatory lesion with necrosis	Pulmonary infection
P33	30	83	Organizing pneumonia	Pulmonary infection
P34	10	63	Fibrous connective tissue; chronic inflammatory cell infiltration in lung tissue	Pulmonary infection
P35	7	49	Blood clots; infiltration of inflammatory cells in lung tissue	Pulmonary infection
P36	2	56	Inflammatory cells with necrosis	Lung abscess
P37	60	63	Lung tissue; partial alveolar wall dust deposition	Pulmonary infection
P48	90	54	Pneumonic lesions with fibrosis and carbon deposition	Lung abscess
P49	30	66	Inflammatory cells infiltration in a few broken fibrous tissue	Pulmonary infection
P51	30	61	Mild hyperplasia of alveolar epithelium; histiocyte proliferation in alveolar cavity and alveolar septum	Pulmonary infection
P58	3650	71	Fibrotic interstitium with edema, hyperemia; no alveolar tissue	Pulmonary infection
P61	7	75	Hyperplastic fibrous tissue with chronic inflammatory cell infiltration; a small amount of bleeding and necrosis	Spherical pneumonia
P70	1000	61	Microstructural hyperplasia in the alveolar cavity and pulmonary interstitium; focal hyaline degeneration and a little chronic inflammatory cell infiltration	Pulmonary infection
P72	365	60	Fibrin-like exudates in the alveolar cavity; alveolar septum thickening with fibrosis and chronic inflammatory cell infiltration	Pulmonary infection
P76	30	55	Fibrous tissue hyperplasia	Pulmonary infection
P84	10	61	Fibrous tissue hyperplasia with chronic inflammatory cell infiltration; organic pneumonia	Lung abscess
P91	150	42	Interstitial fibrosis, histiocyte and lymphocyte infiltration	Lung abscess
P92	1	45	Fibrous tissue with lymphocyte and plasma cell infiltration	Pulmonary infection
P93	270	67	Two small pieces of coagulative necrosis; Pulmonary interstitial fibrous tissue hyperplasia with alveolar epithelial hyperplasia; PAS (-); Acid-fast staining (-); Silver staining (-)	Pulmonary infection
P99	20	73	Fibrous tissue cells, scattered and small clusters of glandular epithelial cells	Lung abscess
P100	1	25	Interstitial fibrous tissue hyperplasia with scattered lymphoid hyperplasia	Lung abscess
P106	365	78	Mild hyperplasia of well-differentiated alveolar epithelium with fibrous tissue hyperplasia; vitreous degeneration and chronic inflammatory cell infiltration; a small amount of fibrous exudation	Pulmonary infection

#### Non-infectious pulmonary disease

Among the 30 patients with non-infectious diseases, mNGS showed false-positive results in 13 cases (Additional file 2). Non-infectious diseases included organizing pneumonia (n = 5), lung cancer (n = 17), lymphoma (n = 3), rheumatoid arthritis (n = 2), granulomatous polyangitis (n = 1), drug-induced pneumonia (n = 1) and allergic alveolitis (n = 1). In one case with a false-positive result by the application of mNGS (*Mycobacterium),* the traditional methods detected *Aspergillus*. This case was finally diagnosed with a lung cancer and the condition improved after chemotherapy. Therefore, it was a false-positive both for the conventional tests and the mNGS.

#### Diagnostic performance of CE and mNGS techniques

Altogether our results show that the sensitivity and specificity, the positive predictive values (PPV) and the negative predictive values (NPV) of traditional examinations in the diagnosis of suspected pulmonary infections were 42.1%, 96.7%, 97.0% and 39.7%, respectively. The sensitivity and specificity of the diagnostic performance by mNGS were 53.9% and 56.7%, respectively. Precisely, the PPV was 75.9% while the NPV was 32.7% (Table 3). There were no significant differences between mNGS and CE concerning pathogen detection in sensitivity. The specificity of CE was significantly higher than that of mNGS (96.7% vs. 56.7%, *p* < 0.05).

**Table 3:** Comparison of diagnostic performance between mNGS and conventional examinations (CE) in identify the pathogen in suspected local pulmonary infection

	Infected, N = 76			Non-infected, N = 30		
	CE +	CE −	Total	CE +	CE −	Total
mNGS +	23	18	41	1	12	13
mNGS-	9	26	35	0	17	17
Total	32	44	76	1	29	30

In the diagnosis of suspected local pulmonary infections, the sensitivity, specificity, positive predictive value (PPV) and negative predictive value (NPV) of CE were 42.1%, 96.7%, 97.0% and 39.7%. Respectively, the sensitivity and specificity of mNGS were 53.9% and 56.7%, while the PPV and NPV were 75.9% and 32.7%. There was a poor agreement between CE and mNGS, and the value of kappa coefficient was 0.269 (95% CI: 0.077 to 0.422)

### Lung abscess

31 cases in our study were diagnosed as lung abscess. The average symptom duration before admission was 97.1 days. Male patients accounted for 80.6%. 22 cases had a definitely proven presence of microorganisms, while false-positive was detected in 1 case only. In this false-positive case, mNGS detected *Aspergillus fumigatus,* while the patient’s condition improved gradually without the administration of anti-fungal therapy, so *Aspergillus fumigatus* was considered as a colonization. The detection rate in identifying the pathogen was 67.7% (21/31) with mNGS compared to 29.0% (9/31) with CE, and this comparison in favour of mNGS had statistical significance (*p ˂0.05*). Polymicrobial infections were found in 10 cases, the most common pathogens were anaerobic species coexisted with *Streptococcus constellatus*. The most frequently isolated anaerobes were *Porphyromonas endodontalis*, followed by *Treponema denticola*, *Parvimonas micra* and *Treponema lecithinolyticum*. The most common monomicrobial infection was *Klebsiella pneumoniae*. Details are showed in Table 4.

**Table 4:** Symptom duration before admission and distribution of pathogens in lung abscess

Patient ID	Age (years)	Symptom duration before admission (days)	Pathogens identify by mNGS	Pathogens identify by Conventional examinations
P1	63	150	*Capnocytophaga sputigena*; *Actinomycetes*	*Actinomycetes*
P3	65	30	*Nocardia*	*Nocardia*
P4	46	210	*Porphyromonas endodontalis*; *Treponema denticola*	Negative
P8	50	60	Negative	Negative
P9	66	90	Negative	Negative
P11	33	6	*Klebsiella pneumoniae*	Negative
P14	80	12	*Klebsiella pneumoniae*	Negative
P18	44	20	*Staphylococcus aureus*	Negative
P22	65	700	*Porphyromonas endodontalis*; *Treponema lecithinolyticum*; *Treponema denticola*; *Parvimonas micra*	Negative
P27	71	700	Negative	Negative
P28	74	60	*Porphyromonas endodontalis;Treponema denticola*	Negative
P29	47	5	*Streptococcus constellatus*	Negative
P30	44	11	*Streptococcus constellatus;Treponema denticola*	*Streptococcus constellatus*
P36	56	2	Negative	Negative
P39	55	30	*Penicillium marneffei*	*Penicillium marneffei*
P44	65	120	*Fusobacterium nucleatum;Streptococcus constellatus*	*Fusobacterium nucleatum*
P45	78	5	*Pseudomonas aeruginosa*	*Pseudomonas aeruginosa (MDRO)*
P48	54	90	*Aspergillus fumigatus (False positive)*	Negative
P50	78	20	Negative	*Actinomycetes*
P53	60	30	*Escherichia coli*	Negative
P57	63	270	*Treponema lecithinolyticum*; *Parvimonas micra*	Negative
P60	73	120	*Treponema lecithinolyticum*; *Parvimonas micra*	Negative
P64	62	15	*Streptococcus constellatus*; *Porphyromonas endodontalis*; *Parvimonas micra*	*Streptococcus constellatus*
P82	58	30	*Fusobacterium nucleatum*	Negative
P84	61	10	Negative	Negative
P88	51	7	*Klebsiella pneumoniae*	Negative
P91	42	150	Negative	Negative
P97	54	30	*Haemophilus influenzae*	Negative
P99	73	20	Negative	Negative
P100	25	1	Negative	Negative
P103	62	6	*Streptococcus constellatus*; *Porphyromonas endodontalis*	*Streptococcus constellatus*

## Discussion

In the present study, 71.7% (76/106) of all enrolled cases were finally diagnosed as pulmonary infectious diseases, among which lung abscess accounted for 40.8% (31/76). After the application of mNGS and CE for pathogens detection in the lung tissues obtained by the CT guided biopsy, 64.5% (49/76) patients showed presence of infectious pathogens. The most common detected pathogens were bacteria, *Mycobacterium *spp. and fungi. The sensitivity and specificity of CE were 42.1% and 96.7%, respectively. The sensitivity and specificity of mNGS were 53.9% and 56.7%, respectively. These results did not have the high sensitivity of mNGS as the one reported by Li et al. [9]. These authors reported a retrospective study of mNGS in the diagnosis of infectious pathogens in lung biopsy tissues in 20 patients. Under their study the mNGS identified the infectious pathogens in 15 out of 20 patients, and the sensitivity of mNGS were 100.0% for bacteria, 57.1% for fungi, when compared to culture method. In our study, mNGS was significantly superior to CE in the detection rate of bacterial (96.3% vs 40.7%) and mixed infections (100% vs 50%), but inferior in the detection of fungal (60% vs 90%) and *Mycobacterium *spp*.* (66.7% vs. 100%) infections with no significant difference.

Pulmonary tuberculosis (TB), which is caused by *M. tuberculosis* (MTB)*,* is quite common in China. Conventional diagnostic methods for pulmonary tuberculosis include culture, GeneXpert TB PCR assay of sputum or BALF, lung tissue specimen, observation of caseous necrotizing granuloma after the pathological examination of the lung tissue. And though GeneXpert TB PCR assay is sensitive and quick (2 h). Zhou et al. showed that mNGS produced a sensitivity of 44% for all active TB cases, which was similar to using the GeneXpert TB PCR (42%), but much higher than the conventional methods (29%) [13]. Their study demonstrated mNGS had a similar diagnostic ability of MTB compared to GeneXpert TB PCR in suspected TB. However, in our study, the detection rate of mNGS for *M. tuberculosis* was not better than the CE.

Pulmonary cryptococcosis without HIV is not rare in China, it can be established by serum CrAg test, culture and histopathologic examination of lung tissues. In our previous study, 70.8% of studied cases had a growth of *Cryptococcus neoformans* in the culture of lung tissues obtained by CT-guided percutaneous lung biopsies [14]. In the present study, mNGS produced false-negative results in 3 cases of pulmonary cryptococcosis, when compared with the conventional examination techniques. Recently, a study by Jin-Min Peng also showed that mNGS had a lower diagnostic accuracy rate for fungal infections (76.7% vs 99.2%, *p* < 0.001), when compared with conventional microbiological tests with BALF, mainly due to the low sensitivity in patients with invasive pulmonary aspergillosis (IPA) [12]. One reason for the difficulty for mNGS to detect MTB and fungi may be that these intracellular bacteria would release fewer extracellular nucleic acids due to the intracellular growth characteristics. Another reason for the negative detection rate by mNGS includes the fact that the vast majority of reads were of human origin in the lung tissues, with only a few reads from infecting pathogens. In future, the metagenomics method would be improved by introducing host DNA depletion without influencing infecting pathogens. Recently, Charalampous presented an optimized Nanopore sequencing-based clinical metagenomics framework for bacterial detection that removed up to 99.99% of the host nucleic acid from the clinical respiratory samples and enabled pathogen and antibiotic resistance gene identification within 6 h [15]. The innovation of mNGS technology will improve the detection rate of mNGS for pathogens or will at least develop a new direction in its applications.

Lung abscess is an old disease, which often occurs due to aspiration of oropharyngeal secretions, and the common causative pathogens include obligate and facultative anaerobic bacteria. The detection of obligate anaerobes requires anaerobic culture conditions and appropriate duration of culturing. Routine aerobic bacterial culture may not be able to identify the true pathogens in lung abscess. In early reports, 37.3–64.3% of patients with lung abscesses had an unknown etiology [16–18]. Compared with conventional methods, molecular diagnostic methods have the advantage in identifying the pathogens in lung abscess. Mukae et al. [19] recently investigated the microbiota of lung abscess with BALF using the molecular methods compared to culture method and found 94.9% (56) positive in 59 BALF samples with PCR analysis, in contrast to only 66.1% positive diagnosed by conventional culture examination. With the PCR technique *Fusobacterium *spp. were the most frequently detected bacteria (23.7%), followed by the *S. anginosus* group (15.3%). The obligate anaerobes were detected in 42.4% by PCR compared to only 13.6% by culture methods of the BALF specimens. In addition to this unprecedented higher detection rate, the molecular diagnostic methods detected mixed bacterial infections in 37 patients (66.1%), compared to only 27.1% with the conventional culture method. In another research aimed to investigate mNGS diagnostic performance in lung abscess samples with osteoarticular infections, mNGS methods identified potential pathogens in all cases (100%), with a significantly lower rate of 48.4% detected by the conventional culture testing [20]. Another study by Zhang HC et al. investigated the impact of mNGS on focal infection diagnosis and compared it to CE. Patients with skin and soft tissue, brain, liver and lung local infection were enrolled. Clinical specimens with purulent infections and non-purulent necrotizing tissue were sent for examination, mNGS showed a diagnostic positive percent of 86.30% compared to 45.21% detected by the culture tests and 57.53% estimated by the conventional methods (*p* < 0.05). Interestingly, *Klebsiella pneumoniae* was the most detected pathogens, followed by MTBC [21].

In our study, the positive mNGS detection rate was higher than the traditional detection methods (67.7% vs 29.0%, *p* < 0.05) in lung abscess. Anaerobic coinfection with *Streptococcus constellatus* and monomicrobial with *Klebsiella pneumoniae* were the most commonly detected pathogens. The most commonly detected anaerobes were *Porphyromonas endodontalis*, *Treponema denticola*, *Parvimonas micra* and *Treponema lecithinolyticum*. One research reported that in the lung abscess the predominant isolates of anaerobic bacteria were gram-negative *Bacteroides fragilis*, *Fusobacterium capsulatum* and *necrophorum*, gram-positive anaerobic *Peptostreptococcus* and *microearophillic streptococci* [22]. We consider that the detected different pathogens may result in different duration of disease, initial empiric antibiotic therapy, etc. A similar result is found in mixed infections in lung abscess. Mixed infections have been recognized as being vital in the pathogenesis of lung abscess, and have been reported in 21–50% of cases [16, 17, 23]. In the present study, mNGS detected mixed infections among 10 (10/22) cases. When mNGS and CE were compared, the conventional examination only found a single pathogen in 5 out of 10 of mixed infections.

It is quite difficult to culture for anaerobic bacteria for the CE, so it is a challenge to determine whether anaerobic bacteria are pathogenic bacteria or normal flora when detected by mNGS. Even more, Hilty et al. showed that in healthy adults the lower respiratory tract consisted of main anaerobes such as those from the genus *Prevotella* using NGS [24]. Besides, *Bacteroides phylum*, *Prevotella*, *Firmicutes*, *Proteobacteria genera* and *Veillonella*, *Fusobacterium*, *Streptococcus*, *Pseudomonas* genera were the main bacteria detected [25]. Therefore, when interpreting mNGS results, the reads, the coverage of bacteria are needed, and clinicians should combine them with clinical, radiographic and laboratory results to make the final decision.

Although with the conventional examination and the mNGS, 27 of our patients were not diagnosed with infectious aetiologies, the histopathologic examination showed infiltration of bacterial cells or organizing pneumonia in the diseased lung tissue. After follow-up, the pulmonary infiltration resolved gradually. The possible cause might be empirical antibiotic treatment before collection of the specimens for testing, or some others factors important for the accuracy of the mNGS methods. In our study, 30 cases of non-infectious diseases were confirmed by histopathology. However, mNGS detected suspected pathogens in 13 cases of non-infectious diseases, all these cases didn’t receive target treatment for microorganisms. The detected microorganisms of mNGS could be due to a variety of factors, such as contaminant pathogenic DNA across samples during mNGS library preparation, low-complexity sequences matching low-quality reads from the sample, mis-annotated species, or contaminants from database entries that also contain reads to human DNA, sequencing adaptors, or vectors, colonization.

There are several limitations associated with this study. First of all, we fail to evaluate the diagnostic value for RNA virus infection, for not apply multiplex PCR for detection of common respiratory virus except for influenza A/B, lack of mNGS RNA sequencing; also, the limited cases for invasive methods to obtain the sample stand as a limitation factor in our studies Then, for a retrospective study, there is a certain selection and recall bias. In addition, there are no unified definite pathogens criteria for mNGS results, so it is yet difficult to distinguish between pathogenic and colonizing microorganisms.

## Conclusions

The most common cause of local pulmonary infection are bacteria, *Mycobacterium* spp. and fungi. The advantages in the diagnostic performance by the mNGS lie in the detection of bacterial and mixed infections in patients suspected with a local pulmonary infection, while the method is not sensitive enough to identify *Mycobacterium* spp. and fungi compared to the conventional examination in lung tissues obtained by a CT-guided biopsy.

## Supplementary Information

**Additional file 1.** Details about 76 cases diagnosed with a pulmonary infectious disease.

**Additional file 2.** Microorganisms detected by mNGS in non-infectious pulmonary disease.

## Data Availability

The dataset used and/or analyzed during the current study are available from the corresponding author on reasonable request.

## Abbreviations

CT: Computerized tomography
mNGS: Metagenomic next-generation sequencing
BALF: Bronchoalveolar lavage fluid
CE: Conventional examinations
PCRs: Polymerase chain reactions
CMV: Cytomegalovirus
PAS: Periodic acid-Schiff staining
GMS: Grocott-Gomori's (or Gömöri) methenamine silver staining
CrAg: *Cryptococcus* Capsular polysaccharide antigen
G test: (1,3)-β-D-glucan test
GM test: Galactomannan test
MTBC: *Mycobacterium tuberculosis complex*
HIV: Human immunodeficiency virus
PPV: Positive predictive values
NPV: Negative predictive values
TB: Tuberculosis
MTB: *Mycobacterium tuberculosis*
IPA: Invasive pulmonary aspergillosis
