# Supplementary material for: Diagnostic performance of the metagenomic next-generation sequencing in lung biopsy tissues in patients suspected of having a local pulmonary infection
Source: BMC Pulm Med. 2022 Mar 29;22:112. doi: 10.1186/s12890-022-01912-4 (PMC8962165; doi:10.1186/s12890-022-01912-4)
Supplement: Supplementary file 2 — Additional file 2. Microorganisms detected by mNGS in non-infectious pulmonary disease. [file 12890_2022_1912_MOESM2_ESM.docx]

Additional file 2. Microorganisms detected by mNGS in non-infectious pulmonary disease.

| Patient ID | Age (years) | Symptom duration before admission (days) | The potential pathogens considered by mNGS | Final diagnosis |
| --- | --- | --- | --- | --- |
| P7 | 74 | 90 | *Epstein-Barr virus* | Diffuse large B-cell lymphoma |
| P40 | 77 | 30 | *Aspergillus* | Lung adenocarcinoma |
| P47 | 62 | 14 | *Epstein-Barr virus* | Organizing pneumonia |
| P62 | 55 | 7 | *Bacteroides fragilis* | Allergic alveolitis |
| P65 | 83 | 1000 | *Klebsiella pneumoniae* | Lung adenocarcinoma |
| P74 | 80 | 700 | *Enterobacter hormaechei* | Secondary malignant tumor of lung |
| P77 | 44 | 60 | *Epstein-Barr virus* | Angioimmunoblastic T-cell lymphoma |
| P80 | 71 | 2500 | *Pseudomonas stutzeri* | Small cell lung cancer |
| P81 | 64 | 5 | *Aspergillus* | Lung adenocarcinoma |
| P83 | 50 | 365 | *Mycobacterium* | Lung adenocarcinoma |
| P86 | 58 | 4 | *Pneumocystis jirovecii* | Lung cancer |
| P87 | 70 | 10 | *Epstein-Barr virus* | Organizing pneumonia |
| P94 | 51 | 6 | *Klebsiella pneumoniae* | Lung adenocarcinoma |
